# Adenosine Kinase on Deoxyribonucleic Acid Methylation: Adenosine Receptor-Independent Pathway in *Cancer* Therapy

**DOI:** 10.3389/fphar.2022.908882

**Published:** 2022-06-01

**Authors:** Hao-Yun Luo, Hai-Ying Shen, R. Serene Perkins, Ya-Xu Wang

**Affiliations:** ^1^ Department of Gastrointestinal and Anorectal Surgery, The Second Affiliated Hospital of Chongqing Medical University, Chongqing Medical University, Chongqing, China; ^2^ Department of Neuroscience, Legacy Research Institute, Portland, OR, United States; ^3^ Integrative Physiology and Neuroscience, Washington State University, Vancouver, WA, United States; ^4^ Legacy Tumor Bank, Legacy Research Institute, Portland, OR, United States; ^5^ Mid-Columbia Medical Center, The Dalles, OR, United States

**Keywords:** DNA methylation, adenosine, receptor-independent pathway, adenosine kinase, ADK isoforms, ADK inhibitor, cancer therapy

## Abstract

Methylation is an important mechanism contributing to cancer pathology. Methylation of tumor suppressor genes and oncogenes has been closely associated with tumor occurrence and development. New insights regarding the potential role of the adenosine receptor-independent pathway in the epigenetic modulation of DNA methylation offer the possibility of new interventional strategies for cancer therapy. Targeting DNA methylation of cancer-related genes is a promising therapeutic strategy; drugs like 5-Aza-2′-deoxycytidine (5-AZA-CdR, decitabine) effectively reverse DNA methylation and cancer cell growth. However, current anti-methylation (or methylation modifiers) are associated with severe side effects; thus, there is an urgent need for safer and more specific inhibitors of DNA methylation (or DNA methylation modifiers). The adenosine signaling pathway is reported to be involved in cancer pathology and participates in the development of tumors by altering DNA methylation. Most recently, an adenosine metabolic clearance enzyme, adenosine kinase (ADK), has been shown to influence methylation on tumor suppressor genes and tumor development and progression. This review article focuses on recent updates on ADK and its two isoforms, and its actions in adenosine receptor-independent pathways, including methylation modification and epigenetic changes in cancer pathology.

## 1 Introduction

The relationship between cancer and DNA methylation was first described by Feinberg and Vogelstein, who revealed that changes in DNA methylation promote the development of invasive colorectal cancer (Feinberg and Vogelstein, 1983). This led to the hypothesis that epigenetic silencing of tumor suppressors promotes carcinogenesis, as well as the finding that reversing this silencing suppresses tumor growth and may prevent tumorigenesis (Feinberg and Vogelstein, 1983). Aberrant DNA methylation has been confirmed to influence the development of numerous human cancers (Nejman et al., 2014; Sun et al., 2014). DNA hypermethylation in cancer cells has been studied most extensively as targeting promoter regions, especially the tumor suppressor genes. The promoter region of tumor suppressor genes is structurally rich in CpG and focal hypermethylation often occurs in its promoter region (López-Moyado et al., 2019), which leads to gene silencing, genomic instability, cell apoptosis, altered DNA repair, and cell cycle control (Wu and Bekaii-Saab, 2012). Hypermethylation inactivates the transcription of tumor suppressor genes, but it does not change the sequence of the gene itself. The methylation process and status can potentially be reversed and regulated.

DNA methylation utilizes methyl from S-adenosylmethionine (SAM). DNA methyltransferase (DNMT) catalyzes DNA methylation by transferring the methyl group from SAM to a target adenine or cytosine at a specific DNA site (Zhao et al., 2015), SAM is thus irreversibly converted to S- adenosylhomocysteine (SAH). SAH is then converted into adenosine and homocysteine (Hcy) by S-adenosylhomocysteine hydrolase (SAHH). Studies showed that increased downstream adenosine product can reversely influent the SAH to Hcy and transmethylation. Blockade of an adenosine metabolic enzyme, adenosine kinase (ADK) results in reduced adenosine removal and causes adenosine accumulation, and also elevates SAH level (Boison et al., 2002); the increased SAH, as a potent inhibitor of all DNMT, allows reversal of aberrant DNA methylation and expression of antioncogene (James et al., 2002).

Of note, adenosine, as an essential biological molecule of life, plays an important role in various aspects of cancer pathology, such as tumor immunity, tissue ischemia, hypoxia, revascularization, and apoptosis (Fishman et al., 2009a; Antonioli et al., 2013). Adenosine can conduct its manipulatory effects via the G protein-coupled four subtypes of adenosine receptors, i.e., adenosine A_1_, A_2A_, A_2_B, and A_3_ receptors (A_1_R, A_2A_R, A_2B_R, and A_3_R) (Fredholm et al., 2005; Jacobson, 2009). The activation of adenosine receptors is primarily determined by the availability of extracellular levels of adenosine. In addition to the aforementioned receptor-*dependent* actions, adenosine also yields receptor-*independent* actions, which rely on metabolic and intracellular levels and the metabolism of adenosine (Boison and Yegutkin, 2019). ADK plays a crucial role in the regulation of both extracellular and intracellular adenosine levels (Jacobson and Reitman, 2020) and adenosine receptor-dependent and independent pathways, in coordination with other adenosine metabolizing enzymes (Boison and Yegutkin, 2019). We will briefly review adenosine metabolism with a focus on the relationship between receptor-independent pathways of adenosine and DNA methylation in cancer.

## 2 Deoxyribonucleic Acid Hypermethylation in Tumor-Suppressor Genes

**FIGURE 1:**
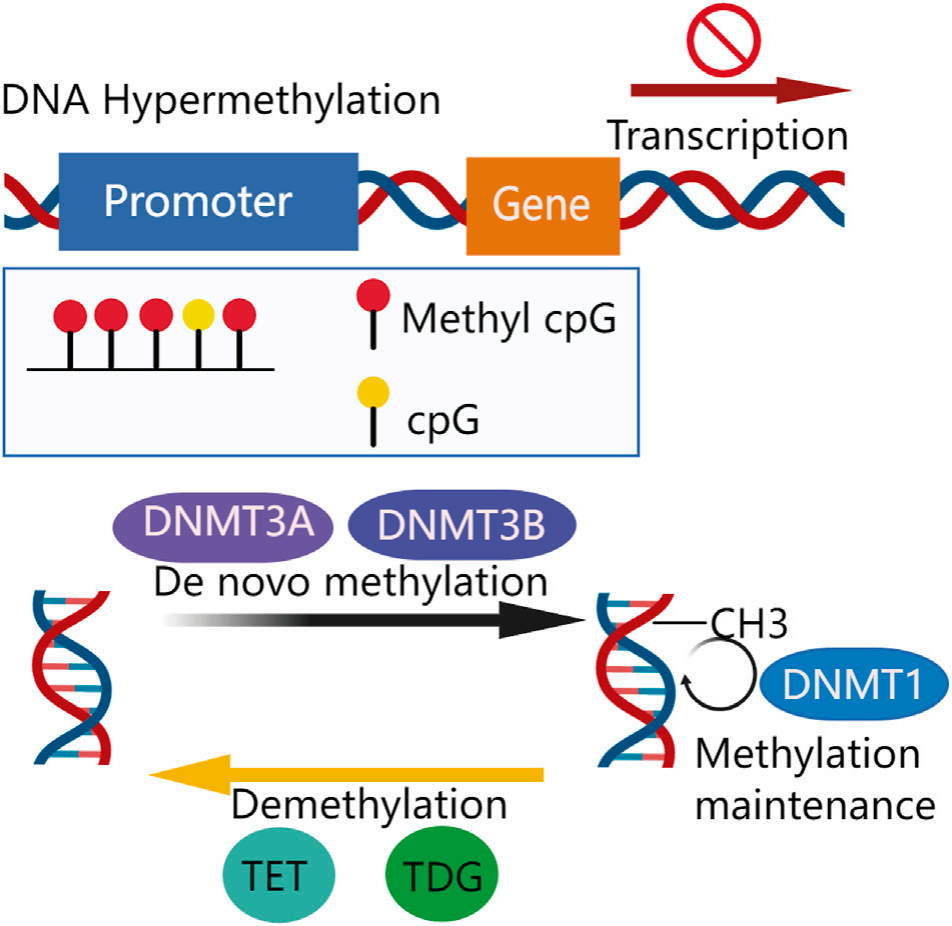
Overview of DNA methylation in CpG of the gene promoter region.

DNA methylation, one of the most abundant epigenetic modifications modulates gene expression and affects cellular processes of metabolism, survival, proliferation, and apoptosis, among others. (Weber et al., 2007; Baylin et al., 2001). Methylation occurs on cytosines within dinucleotide CpG islands (CGIs) which are rich in CpG and usually located at the promoter regions of genes (Oates et al., 2006). It is commonly associated with a transcriptionally repressed status. However, methylation-dependent transcriptional changes can result in both gain and loss of function depending on the gene region affected (Weber et al., 2007). DNA methylation consists of two functionally overlapped aspects: *de novo* and maintenance methylation. A new DNA methylation commonly yields 5-methylcytosine (5-mC), which is established by transferring the methyl group from S-adenosylmethionine (SAM) to cytosine at a CpG site by DNA methyltransferases DNMT3A and DNMT3B (Egger et al., 2006; Hung and Shen, 2003). DNMT3A and DNMT3B mediate *de novo* DNA methylation that does not require a DNA template with preexisting methylation (Okano et al., 1999) whereas DNMT1 contributes to maintaining methylation that involves replicating methylation patterns into a newly-synthesized DNA strand (Goyal et al., 2006). On the other hand, a demethylation system also exists, which includes ten-eleven translocation methylcytosine dioxygenases (TETs) and thymine DNA glycosylase (TDG)-base excision repair (BER) (Pan et al., 2017). The TETs catalyze the oxidation of 5-methylcytosine to 5-hydroxymethylcytosine, and its downstream oxidation products: 5-formylcytosine and 5-carboxylcytosine, are removed by TDG of BER (Figure 1). Both methylation and demethylation systems contribute to the dynamically balanced methylation status of the genome (Weber et al., 2007).

*De novo* methylation is mediated by DNMT3A and DNMT3B to transfer methyl group (-CH3). Methylation is maintained by DNMT1. Demethylation of DNA is mediated by TET, TDG, and BER. A certain extent of promoter CpG island methylation impairs transcription, silencing gene expression.

Alternation in DNA methylation patterns is of importance in cancer pathology without affecting genome editing (Feinberg and Tycko, 2004) while DNA both hypermethylation and hypomethylation are seen in cancers (Das and Singal, 2004; Franco et al., 2008; Sinčić and Herceg, 2011). Cancer-associated methylome alterations are attributable to expressional changes of DNMTs (Morey et al., 2006; Gao et al., 2013; Micevic et al., 2017), which can result in increased genomic instability, expression of oncogenes, and/or decreased expression of tumor suppressor genes (Zhang et al., 2017; Valencia and Kadoch, 2019). Specifically, hypomethylation commonly occurs in oncogenes during cancer development and has been extensively reviewed (Mendizabal et al., 2017); in contrast, DNA hypermethylation is mostly found in tumor suppressor genes (Su et al., 2018). In the present review, we will focus on hypermethylation of tumor suppressor genes and possible adenosine regulations.

Hypermethylation resulting in epigenetic silencing was first demonstrated in the studies of retinoblastoma patients, in which hypermethylation was discovered in the promoter of the retinoblastoma tumor-suppressor *(RB1)* gene (Greger et al., 1989). Since then, a large number of tumor-suppressor genes have been identified as being silenced by DNA hypermethylation in tumorigenesis of different cancers. In colorectal cancers: 1) a cytokinesis-related gene Septin9 was identified highly correlated with the occurrence and development of colorectal cancer (Tanaka et al., 2002) and DNA methylation is the main mechanism regulating Septin9 gene expression (Sellin et al., 2011; Connolly et al., 2011), which mediates cytokinesis failure, leading to aneuploidy, centrosome amplification, and multipolar mitosis, eventually cause cell division and carcinogenesis (Sun et al., 2019; Cortez et al., 2016). In addition, the methylation level of the Septin9 gene is also considered to have clinical guiding significance due to the correlation with malignancy (Sun et al., 2019; Bae et al., 2017) and the overall survival of patients (Yang et al., 2019). Methylation of Septin9 in peripheral blood is the first blood DNA methylation marker approved by the US Food and Drug Administration (FDA) for CRC screening (Church et al., 2014), and is now widely used as a colorectal cancer biomarker (Xie et al., 2018). 2) MLH1, as the homolog of MutL, the main protein of the mismatch repair (MMR) system (Gelsomino et al., 2016), is silenced due to the hypermethylation of its promoter (Liu et al., 2017), resulting in deficient mismatch repair (dMMR) (Yamamoto and Imai, 2015). The replication errors of microsatellites (MS) cannot be corrected and accumulate continuously, resulting in microsatellite instability (MSI). Significance correlations were found in MLH1 promoter methylation and gender, tumor position, tumor differentiation, MSI, MLH1 protein expression, and v-RAF murine sarcoma viral oncogene homolog B1(BRAF) mutation in CRC patients (Li et al., 2013). In gastric cancer: runt-related transcription factor 3 (RUNX3) is an important downstream target of transforming growth factor-beta (TGFb) superfamily signaling, CpG silencing in the promoter region of regulated genes by hypermethylation is thought to be one of the mechanisms leading to loss of gene function (Fan et al., 2011). Through the detection of plasma samples, RUNX3 methylation level was considered to be a risk factor for gastric cancer metastasis and a potential indicator of gastric cancer progression (Fan et al., 2011). In breast cancer, the following genes are described: 1) ataxia-telangiectasia mutation (ATM) gene, a tumor suppressor plays a crucial role in maintaining genome integrity by activating cell cycle checkpoints and promoting the repair of DNA double-strand breaks (Wengner et al., 2020). Hypermethylation in ATM gene promoter downregulates ATM mRNA expression and positively correlates with increased tumor size and advanced disease stages III and IV (Begam et al., 2017; Cao et al., 2018). 2) a DNA repair gene, breast cancer 1 (BRCA1) - when a pathogenic mutation occurs, resulting in homologous recombination deficiency, the damaged DNA is difficult to repair, and it has been proved to easily lead to malignant tumors such as triple-negative breast cancer (TNBC) (Sharma, 2016). By comprehensively comparing the molecular biological characteristics of TNBC patients with BRCA1 hypermethylation and BRCA1 mutation, Dominik Glodzik et al. found the frequency of BRCA1 promoter hypermethylation correlates with clinicopathological variables, molecular subtypes, and patient outcomes in the early-stage of TNBC. This study indicated hypermethylation of the BRCA1 promoter region as a potential biomarker of early TNBC occurrence (Glodzik et al., 2020).

Together, the evidence indicates that DNA hypermethylation in the promoter region of tumor suppressors plays a crucial role in tumorigenesis, which is an epigenetic hallmark of various types of cancer. Table 1 lists representative tumor suppression genes with hypermethylation in their promoters. Indeed, the demethylation treatment strategy was proposed after discovering abnormal hypermethylation in tumors and researchers started the attempt to reverse hypermethylation (Issa, 2007). 5-Aza-2′-deoxycytidine (5-AZA-CdR, decitabine) (Karahoca and Momparler, 2013) was shown to have the ability to reverse DNA methylation, activate tumor suppressor genes, and promote apoptosis (Flohr and Breull, 1975), with possible mechanisms relied on the inhibition of DNMT1 (Chen et al., 2019). In a xenograft mouse model bearing the colon cancer line, HCT116, the 5-AZA-CdR was shown to demethylate the *CDH13 gene*, restoring its expression, resulting in a suppression of tumor growth (Ren and Huo, 2012). However, related experiments confirmed that gene re-expression in response to 5-AZA-CdR was transient and re-silenced upon drug removal (Bender et al., 1998; Egger et al., 2007). Besides, studies have also pointed out that 5-AZA-CdR treatment has always been interpreted with caution since the 5-AZA-CdR treatment can non-selectively affect the entire genome (Christman, 2002; Sigalotti et al., 2014). The non-selective demethylation yielded from 5-AZA-CdR may trigger serious adverse reactions, which limit its clinical use. Thus, methylation inhibitors with fewer side effects and higher selectivity on cancer cells are of interest for development.

**TABLE 1:** Promoter hypermethylated genes in cancers.

*Cancer* Type	Gene	Detection	Hypermethylation Indication	References
Colorectal cancer	Septin9	Peripheral blood assays	Tumor malignancy	(Sun et al., 2019; Yang et al., 2019)
			Affect overall survival of patients	
	MLH1	Immunohistochemistry (indirect)	Tumor differentiation and position	Li et al. (2013)
			BRAF mutation	
Gastric cancer	RUNX3	Peripheral blood assays	Tumor differentiation	Fan et al. (2011)
			Risk factors for the carcinogenesis of chronic atrophic gastritis with *H. pylori* infection	
			Tumor malignancy	
Lung cancer	SHOX2	Bronchial aspirates Peripheral blood assays	Early detection of lung cancer with high sensitivity and specificity	Kneip et al. (2011)
Breast *Cancer*	APC	Peripheral blood assays	Better sensitivity than traditional tumor markers for early detection of breast cancer	(Van der Auwera et al., 2009; Swellam et al., 2015; Debouki-Joudi et al., 2017)
	BRCA1	Peripheral blood assays	Biomarkers of early TNBC occurrence	(Sharma, 2016; Winter et al., 2016)
Prostate *Cancer*	CDH13	Peripheral blood assays	Increased risk of death Independent predictor of a poor prognosis	Wang et al. (2014)

## 3 Adenosine Regulations in Cancer

Adenosine is an endogenous purine nucleoside and an intermediary metabolite in DNA methylation. Adenosine accumulation has been observed in tumor tissues, which is associated with tumor growth, invasion, metastasis, and immune evasion in tumor pathology (Mastelic-Gavillet et al., 2019; Borodovsky et al., 2020; Wang et al., 2021). Adenosine has immunosuppressive effects on intratumoral immune populations (Stagg and Smyth, 2010). It can bind cell surface receptors and is secreted in a paracrine or autocrine manner or reverse regulate DNA methylation through substrate accumulation, thus exerting its biological effect. Major pathways regarding adenosine production, metabolic removal, and transportation across the cell membranes have been extensively reviewed otherwise (Boison and Yegutkin, 2019), we briefly summarize them as follows.

Adenosine production and transportation in cancer tissues are similar to physiological conditions; extracellular ATP and ADP can rapidly metabolize to adenosine monophosphate (AMP) majorly through two steps of dephosphorylation: 1) The first step, ATP and ADP are both converted to AMP by ecto-nucleoside triphosphate diphosphohydrolase-1 (CD39); then 2) AMP can generate adenosine by the final dephosphorylation reaction catalyzed by the enzyme ecto-5′-nucleotidase (CD73) (Fishman et al., 2009b) - this called CD39/CD73 pathway. Alternatively, cyclic ADP ribose hydrolase (CD38) can convert adenosine diphosphate ribose (ADPR) to AMP, this process can be regulated by ecto-nucleotide pyrophosphatase/phosphodiesterase 1, NPP1 (CD203a) (Gazzoli et al., 2002; Häusler et al., 2011). Afterward, CD73 converts AMP into adenosine–called CD38/CD203a pathway. In adenosine transportation across membranes, equilibrative nucleoside transporter (ENT) and concentrative nucleoside transporters (CNTs) play important roles (Song et al., 2017); Adenosine removal differs between intracellular and extracellular. Extracellular adenosine is converted to inosine by adenosine deaminase (ADA), which is widely expressed in the plasma as well as on the cell membrane. Inosine is then derivatized (removed from ribose) by purine nucleoside phosphorylase (PNP), which converts it to hypoxanthine. It is worth noting that ADA not only metabolizes adenosine, it also allosterically modulates ARs, resulting in a positive effect of amplifying downstream signals (Borea et al., 2018) including 1) enhanced AR1 sensitivity to adenosine (SU Xiaoyang, 2018); 2) interaction of ADA-CD26 complex in T cells with ADA-anchored protein in dendritic cells enhanced T cell proliferation (Pacheco et al., 2005), etc.

**FIGURE 2:**
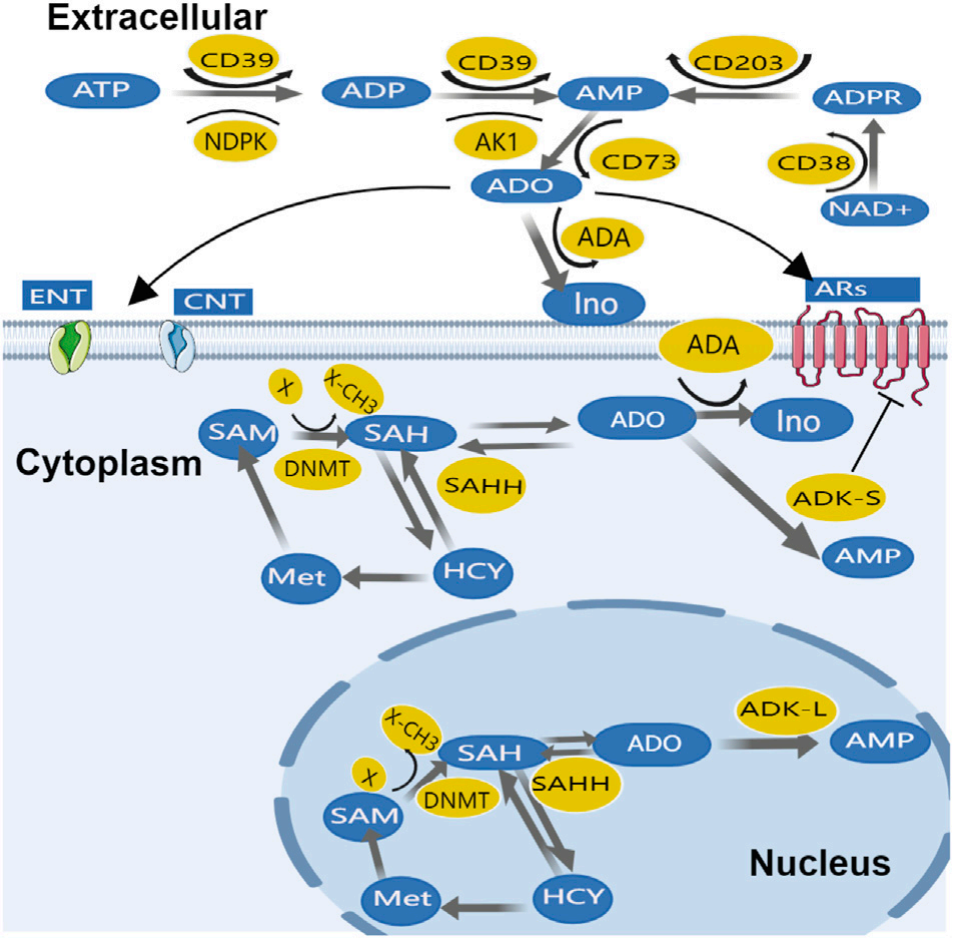
Major pathways of adenosine production, metabolism, and transport.

While the metabolism of intracellular adenosine is mainly dominated by ADK. The major adenosine removal enzyme ADK has two isoforms with distinguished subcellular expression patterns; while ADK short isoform (ADK-S) is expressed dominantly in cytosolic space, ADK long isoform (ADK-L) is solely located in the nuclei (Cui et al., 2009; Fedele et al., 2005). Intracellular adenosine is mainly removed by ADK-S, which converts adenosine to AMP (Boison and Yegutkin, 2019). Adenosine can also be directly inactivated on the cell surface by adenosine deaminase (ADA). In addition, adenosine metabolism also depends on adenosine phosphoribosyltransferase (APRT) to catalyze adenine reaction with ribose 1-phosphate to generate phosphate and adenosine in the nucleus. However, when energy consumption increases and/or energy supply is compromised, ATP is converted into AMP by adenylate kinase-1 (AK1) and nucleotide diphosphate kinase (NDPK), and then dephosphorylated into adenosine by 5-nucleotidase (Eltzschig et al., 2012). This process promotes extracellular ATP regeneration through a reversible phosphonate transfer reaction (Boison, 2013). The nucleoside transporters and adenosine removal enzymes maintain a dynamic balance between extracellular and intracellular adenosine (Figure 2). Due to mitochondria being the main source of ATP, mitochondrial bioenergy is related to adenosine homeostasis (Ashar et al., 2017).

Moreover, adenosine metabolism is a part of the transmethylation pathway, in which DNA can be methylated by DNMTs while SAM donates methyl group (-CH3) via a methyltransferase (MT) - catalyzed transmethylation reaction (Figure 2). Then, the SAM converted SAH is hydrolyzed to adenosine and Hcy by SAHH. Interestingly, the nuclear form of ADK-L drives methyl flux, enhancing DNA and histone methylation (Yegutkin, 2014).

Extracellular adenosine turnover is mediated by AR, ENT, and CNT. Factors that mediate adenosine production and removal include the enzymes CD39, CD73, ADK, and ADA. Additionally, intracellular adenosine metabolism depends on the cytoplasmic form of ADK-S and ADA. In the nucleus adenosine is part of the transmethylation pathway in which DNA is methylated by DNMT. ADK-L participates in driving the methyl groups through the transmethylation pathway affecting DNA and histone methylations. For the sake of clarity, only the most important enzymes are mentioned.

## 4 Adenosine Receptor-Dependent Pathway in Cancer

Substantial evidence indicates that adenosine mediates its physiological effects (Borea et al., 2018) as well as its pathophysiological actions in cancer (Fishman et al., 2009a; Franco et al., 2021) through the activation of four adenosine receptors (ARs), i.e., A_1_R, A_2A_R, A_2B_R, and A_3_R. Activation of ARs by specific ligands, agonists, or antagonists will regulate the occurrence and development of tumors through a series of signaling pathways (Borea et al., 2018; Franco et al., 2021). A_1_R has been studied mainly in glioblastoma (Synowitz et al., 2006; Fishman et al., 2009a), where A_1_R activation on microglia/macrophages in the tumor suppresses not only the production of cytokines such as interleukin-1β but also stromal metalloproteinase (MMP) (Tsutsui et al., 2004). Based on that, A_1_R is thought to have the effect of inhibiting tumor growth (Synowitz et al., 2006). Besides, what cannot be ignored is the important role of ARs in tumor immunity. In the tumor microenvironment, adenosine suppresses antitumor immunity, essentially through A_2A_R and A_2B_R (Buisseret et al., 2018). In particular, the A_2A_R, due to the high concentration of Ado in the tumor microenvironment, activates Gs-coupled A2AR and leads to an increase in cAMP, thereby inhibiting the activation of tumor lymphocytes (Fishman et al., 2009a; Merighi et al., 2019). Therefore, selective antagonism of A_2A_R can reduce cAMP levels, thereby enabling lymphocytes to effectively fight tumor cells (Franco et al., 2021). So far, a large number of clinical trials on A_2A_R/A_2B_R antagonists are also in progress (Franco et al., 2021). On the other hand, adenosine was observed to increase HIF1α protein accumulation under hypoxia situations through cell surface A_3_R interaction in various tumors (Merighi et al., 2005), and HIF1α plays an important role in tumor VEGF expression and angiogenesis (Merighi et al., 2005). Based on the relationship between tumor, hypoxia, and adenosine concentrations, A_3_R antagonists are considered to have a potential role in cancer therapy (Franco et al., 2021). Adenosine receptor-dependent pathway in cancer was already described in detail by Pier Andrea (Borea et al., 2018).

## 5 Adenosine Receptor-independent Pathway With Deoxyribonucleic Acid Methylation in Cancer

As an ATP metabolite, adenosine is released by all cell types and is shown to accumulate in tumor cells, which is associated with increased angiogenesis, high metabolism rate, and compromised hypoxia of the microenvironment (Losenkova et al., 2020). Accumulation of adenosine in the tumor microenvironment (TME) (de Lera Ruiz et al., 2014) has been proven to play an important role in tumor immunity, high concentrations of adenosine inhibit tumor immune effects (Ohta and Sitkovsky, 2001; Ohta et al., 2006; Ohta, 2016) and facilitate angiogenesis (van de Veen et al., 2020), which offers the possibility of targeting adenosine in cancer pathology and manipulation of adenosine actions represents a potential anti-cancer strategy. Meanwhile, solid tumors can maintain adenosine gradients - the adenosine levels in the tumor center are higher than in the peripheral area of the tumor (Ohta et al., 2006). High levels of adenosine are shown to hinder tumor growth and proliferation. For instance, peripheral tumor cells located in the parenchyma and stroma have been shown to have high proliferative and invasive abilities (Seetulsingh-Goorah, 2006) and their proliferation can be suppressed by adenosine (Seetulsingh-Goorah, 2006; Schiedel et al., 2013). Based on that, Sanna S. Virtanen *et al.* found adenosine with relatively high (10 μmol/L for the former and 50 μmol/L for the latter) concentrations showed the ability to inhibit tumor invasion and migration (Schiedel et al., 2013). Besides, incubation of human prostate carcinoma cell line PC-3 cells triggered a concentration-dependent increase in cAMP levels with increasing adenosine concentrations. However, in the presence of A_2B_R-selective antagonists, no changes in cAMP levels were observed (Schiedel et al., 2013). In addition, in a study on glioblastoma, Helena Marcelino *et al.* found that proliferation/viability of glioblastoma cells was significantly reduced after 30 μM doses of adenosine for three consecutive days. At the same time, the cocktail of adenosine receptor antagonists (Fredholm et al., 2001) was administered, but the tumor suppressor effect was not affected (Marcelino et al., 2021).

The above described discrepant effects of adenosine on pro-and anti-tumor cell growth suggest a possible involvement of multiple mechanisms. In other words, its inhibitory effect on proliferation is proposed beyond receptor-mediated adenosine activity (Virtanen et al., 2014), though the underlying mechanisms remain unclear. Possible metabolic contributors that determine high-adenosine level mediated inhibition may include extracellular adenosine deaminase activity, subsequent cellular uptake, interconversion of transported nucleosides, simultaneous inhibition of multiple protein kinases (Virtanen et al., 2014), as well as ADK actions. However, the potential involvement of multiple pathways in adenosine production, transportation, and metabolism, suggests the complexity of adenosine’s effect on tumor pathology.

Importantly, the metabolism of adenosine also affects the methylation process. When SAM/SAH is an important source of adenosine, it can reverse regulate DNA methylation through the substrate accumulation effect (Kloor and Osswald, 2004; Viré et al., 2006). Kai X *et al.*, by observing the effects of different concentrations of adenosine (0, 1.5, 3.0, 4.5 mmol/L) and treatment time (24, 48, 72, 96 h) on the proliferation, apoptosis, and HMLH1 expression of human colorectal cancer cell SW480, found that after treating colorectal cancer cells with different concentrations of adenosine, the hypermethylation of tumor suppressor genes hMLH1 was reversed and inhibited the proliferation of tumor cells. This kind of positive effect increased with the addition of exogenous adenosine concentration and treatment time (Xie et al., 2014). Meanwhile, Li Q *et al.* found that after treating human colorectal cancer cells SW480 with adenosine (3.0 mmol/L) for 72 h, the activity of methyltransferase (DNMT1 and DNMT3A) in these cells was inhibited, and similar to the above finding the hypermethylation of tumor suppressor genes RECK was reversed (Li et al., 2015). Like the aforementioned, alternations in DNA methylation patterns impact the occurrence and development of tumors (Klutstein et al., 2016). Studies regarding adenosine and DNA methylation status have also been reported in non-tumor disorders such as epilepsy, showing that inhibition of DNA methyltransferase activity during adenosine release is associated with restoration of global DNA methylation levels (Williams-Karnesky et al., 2013), this suggests that adenosine manipulation is a potential strategy in cancer manipulation via DNA methylation.

However, side effects such as flushing, dyspnea, chest pain, hypotension, bradycardia, etc. make the usage of exogenous adenosine less feasible for cancer treatment (Pritchard et al., 2010; Galagudza et al., 2012; Gul et al., 2020). A further question is whether systemic adenosine leads to a reversal of global methylation status or affects the site that should have been hypomethylated. Another concern is adenosine receptor-mediated action showed a cancer-promoting effect. Conversely, accumulating evidence supports ADK as a therapeutic target in cancer (Boison and Yegutkin, 2019; Murugan et al., 2021). The expression of ADK was shown to be upregulated in specific cancer types, including colorectal cancer (Giglioni et al., 2008), and breast cancer (Wang and Yang, 2014; Shamloo et al., 2019). Most recently, it has been found that a significantly enhanced expression of ADK in specimens of patients with glioma, both the tumor center and peritumoral tissue (de Groot et al., 2012). The general increase of purine metabolizing enzymes including ADK may allow accelerated purine metabolism to support the growth of cancer (Vannoni et al., 2004; Giglioni et al., 2008).

## 6 Targeting Adenosine Kinase on Deoxyribonucleic Acid Methylation in Cancer

The above described receptor-independent pathway mechanisms of adenosine play important roles in various types of cells with diverse functions (Boison et al., 2002). As an essential adenosine removal enzyme, inhibition of ADK can be more effective to decrease the cellular reuptake of adenosine and thereby increase the ambient concentration of extracellular adenosine (Newby et al., 1983; Davies et al., 1984). ADK inhibition was hypothesized to function as a site- and event-specific modulator for adenosine levels (Yamamoto and Imai, 2015; Cortez et al., 2016). This also provides a new direction for the treatment of tumors–targeting overexpression of ADK to regulate onsite adenosine level and DNA methylation, thereby affecting the proliferation and apoptosis of tumor cells. ADK-based adenosine intervention can avoid the aforementioned side effects of systemic adenosine administration (Liu et al., 2019) and pharmacokinetics limitation of the very short half-life in circulation (Hwang et al., 2016). ADK inhibitors have been revealed to have anti-inflammatory, antinociceptive, and anticonvulsant features (McGaraughty et al., 2005), and is being considered for the treatment of various diseases, including diabetes (Annes et al., 2012) and diseases of the nervous system (Chen et al., 2016).

ADK inhibitor development was initially based on 5-iodotubercidin (5-ITU), and 5′-amino-5′-deoxyadenosine (Cottam et al., 1993; Wiesner et al., 1999; Chen et al., 2016). Since then, several types of ADK inhibitors have been developed, which are classified as nucleoside and non-nucleoside ADK inhibitors (Boison, 2013). Nucleoside ADK inhibitors are adenosine derivatives that have hydroxylated ribose or cyclopentane rings, and additional purines or pyrimidine heterocyclic bases (Gomtsyan and Lee, 2004). The 5-aza group of the purine ring is replaced by a carbon connected to iodine. These compounds bind to enzymes to competitively inhibit adenosine (McGaraughty et al., 2005). In contrast, non-nucleoside ADK inhibitors lack ribose or cyclopentane rings, while some of them are constructed on pyrimidine or pyridyl pyrimidine nuclei. The non-nucleoside ADK inhibitors have been shown to relieve pain and inflammation in animal models (McGaraughty et al., 2005). Some ADK inhibitors are based on 6-(het)aryl-7-deazapurine pro-nucleotides that can inhibit cell growth by strongly inhibiting ADK activity (Spácilová et al., 2010), however, the mechanism of this finding has not been further investigated. Helena Marcelino *et al.* tested the effect of two ADK inhibitors on tumor cells in experiments on glioblastoma, and the results suggested that both ITU (25 μM) and ABT702 (15 μM) affected cells proliferation/viability (Marcelino et al., 2021). Co-incubation of ITU (25 μM) and adenosine (30 μM) produced a strong and similar decrease in cell proliferation in both GBM cell lines compared to ITU alone, this suggests that only 25 mM ITU may be sufficient to generate the maximum accumulation of intracellular adenosine (Marcelino et al., 2021). Zhang LM *et al.* showed that 5-ITU with concentrations (1, 2, 4, 6, 8, 10 μmol/L) for 48 h could significantly inhibit proliferation and induced apoptosis in a colon cancer cell line HT-29 (Zhang and Xie, 2015). Compared to the inhibitory effect of each concentrations group on HT-29 cells, the 6 μmol/L group showed a better effect on HT-29 cells, and the tumor suppressor gene DLC-1 in HT-29 cells was up-regulated and its methylation level was decreased after being treated with 2, 4, and 6 μmol/L ITU, respectively, this effect increases with increasing concentration (Zhang and Xie, 2015). As discussed above, ADK may play a potential adenosine receptor-independent epigenetic function, however, current available ADK inhibitors have not yet been reported to have high selectivity to target ADK-L or ADK-S. To distinguish the role of ADK-S and ADK-L on the regulation of cytoplasmic or nuclear adenosine levels and their possible epigenetic functions, using genetic approaches may bring us the answer.

Targeted therapy is a new strategy for cancer treatment. The goal is to use gene therapy to suppress the endogenous expression of ADK, with or without selectively targeting its two isoforms, i.e., the nuclear ADK-L and cytosolic ADK-S (Chen, 2010). Previous studies identified two independent promoters driving the expression of ADK isoforms, suggesting that each of the two isoforms of ADK are independently regulated at the transcriptional level (Cui et al., 2011), and independent transcriptional regulation may in turn indicate distinct physiological functions of the two isoforms (Boison, 2013). Besides, distinguish expression locations of two isoforms indicate that ADK-L (vs. ADK-S) has a unique role in proliferation and differentiation - two main nuclear activities associated with cancer pathology (Cui et al., 2009; Kiese et al., 2016). In patients with grade II and III gliomas, both subtypes of ADK are increased in the tumor and peritumoral areas, in addition to the detection of tumor invasion in the peritumoral tissue suggesting that ADK is involved in glioma progression and ADK level elevations may be associated with epilepsy in glioma patients (Huang et al., 2015). Amir E *et al.* reported a high positive correlation between ADK-L expression and whole-genome methylation in HeLa cells, (Wahba et al., 2021). Most recently, Shen HY *et al.* revealed that the expression level of ADK-L in breast cancer tissue was elevated compared to adjacent tissues, while the ADK-S expression level had no significant change, by measuring the protein expression level (Shamloo et al., 2019). Selective knockout of ADK isoforms via CRISPR/Cas9-mediated approaches suppressed breast cancer cell migration and invasion, which with the elevation of a tumor-related enzyme, matrix metalloproteinases, and downregulation of cyclin D2 and THB1 (Shamloo et al., 2019). Williams Karnesky *et al.* transfected ADK deficient BHK-AK2 cells with ADK-L- or ADK-S-expressing plasmids (Williams-Karnesky et al., 2013). ADK-L receptors showed a 400% increase in overall DNA methylation compared to controls, while ADK-S receptors showed only a modest 50% increase in overall DNA methylation. While both isoforms of ADK are involved in the regulation of overall DNA methylation, the nuclear subtype is more effective in regulating DNA methylation (Williams-Karnesky et al., 2013). ADK-L affects epigenetic remodeling by regulating methyltransferase activity and is considered the preferred mechanism for adenosine clearance in the nuclei (Boison and Yegutkin, 2019). ADK-L is directly related to the S-adenosylmethionine-dependent transmethylation pathway, which drives DNA and histone methylation (Boison, 2013). ADK-S regulates extracellular adenosine concentration for the availability of ARs activation (Pignataro et al., 2007; Boison and Yegutkin, 2019).

These studies support the observed functional differences of ADK-L and ADK-S in cancer. While ADK-L and ADK-S control adenosine concentrations in the nucleus and cytoplasm/extracellular respectively, ADK-L may play a role in adenosine receptor-independent regulation of epigenetic functions, and ADK-S determines adenosine availability for activation of adenosine receptors (Pignataro et al., 2007; Williams-Karnesky et al., 2013). Additional experimental evidence is needed to evaluate this notion. Together, selective inhibition of ADK-L is indicated as a novel adenosine receptor-independent strategy to offer a new perspective on cancer therapy, which may achieve more precise cancer intervention than general ADK or ADK-S manipulation.

## 7 Prospect and Challenge

With the observations that ADK inhibitions with isoform- and site-selective manners enhance the beneficial effect of endogenous adenosine and avoid various side effects of systemic manipulation of adenosine and adenosine receptors, research on ADK has made considerable progress in recent years. The emergence of new molecular tools including genetic approaches has enabled deeper exploration of ADK function. Further characterization of the metabolism of adenosine in different subcellular contexts, including cytoplasm, nucleus, and extracellular space, is needed for potential targeted ADK therapy. Studies have shown that elevated adenosine levels are related to apoptosis in various cancers (Xie et al., 2014; Jafari et al., 2017), which may be attributed to nuclear ADK-L (vs. ADK-S). In addition, the ADK effects on epigenetics, especially DNA methylation, may also be through its direct interaction with other nuclear proteins (Wang et al., 2005; Mohannath et al., 2014) rather than its regulation on the adenosine level. We should always bear in mind the challenge that increased adenosine levels can: 1) inhibit immune and inflammatory responses; 2) stimulate angiogenesis: epigenetic regulation of pro-angiogenic genes by ADK, and is thought to be another mechanism by which ADK is involved in cancer (Murugan et al., 2021). Knockdown of ADK decreases the methylation level of the VEGFR2 promoter region, which elevates intracellular adenosine and promotes proliferation, migration, and angiogenesis of human endothelial cells (Xu et al., 2017)—all aspects that may promote tumor growth. Last but not least, the downregulation of ADK found in hepatocellular carcinoma cells (YH, 2017) suggests the diversity of ADK changes across cancers. In summary, additional studies are needed to fully understand the role of adenosine in cancer pathology and to reveal the anticancer potential of ADK inhibition.
