# Corrigendum: Adenosine kinase on deoxyribonucleic acid methylation: Adenosine receptor-independent pathway in cancer therapy

**DOI:** 10.3389/fphar.2023.1200491

**Published:** 2023-04-12

**Authors:** Hao-Yun Luo, Hai-Ying Shen, R. Serene Perkins, Ya-Xu Wang

**Affiliations:** ^1^ Department of Gastrointestinal and Anorectal Surgery, The Second Affiliated Hospital of Chongqing Medical University, Chongqing, China; ^2^ Department of Neuroscience, Legacy Research Institute, Portland, OR, United States; ^3^ Integrative Physiology and Neuroscience, Washington State University, Vancouver, WA, United States; ^4^ Legacy Tumor Bank, Legacy Research Institute, Portland, OR, United States; ^5^ Mid-Columbia Medical Center, The Dalles, OR, United States

**Keywords:** DNA methylation, adenosine, receptor-independent pathway, adenosine kinase, ADK isoforms, ADK inhibitor, cancer therapy

In the published article, there was an error in the **Affiliation** for Authors Hao-Yun Luo and Ya-Xu Wang. Instead of “^1^Chongqing Medical University, Chongqing, China, ^2^Department of Gastrointestinal and Anorectal Surgery, The Second Affiliated Hospital of Chongqing Medical University, Chongqing Medical University, Chongqing, China”, the correct affiliation is “^1^Department of Gastrointestinal and Anorectal Surgery, The Second Affiliated Hospital of Chongqing Medical University, Chongqing, China”.

The authors apologize for this error and state that this does not change the scientific conclusions of the article in any way. The original article has been updated.

